# The Spread of Rabies Among Dogs in Pranburi District, Thailand: A Metapopulation Modeling Approach

**DOI:** 10.3389/fvets.2020.570504

**Published:** 2020-11-19

**Authors:** Panchiwa Komol, Sawitri Sommanosak, Parima Jaroensrisuwat, Anuwat Wiratsudakul, Kansuda Leelahapongsathon

**Affiliations:** ^1^Department of Clinical Sciences and Public Health, Faculty of Veterinary Sciences, Mahidol University, Nakhon Pathom, Thailand; ^2^Monitoring and Surveillance Center for Zoonotic Diseases in Wildlife and Exotic Animals, Faculty of Veterinary Science, Mahidol University, Nakhon Pathom, Thailand; ^3^Department of Veterinary Public Health, Faculty of Veterinary Medicine, Kasetsart University, Kamphaeng Saen, Nakhon Pathom, Thailand

**Keywords:** metapopulation, rabies, SEIR model, spatial analysis, Thailand

## Abstract

Rabies, a deadly zoonotic disease, is causing serious public health problems worldwide. Dogs are considered the main reservoir for rabies infection in humans. A better understanding of the dissemination of rabies in the dog population is crucial. The present study, therefore, aimed to explore the subpopulation of dogs roaming around rabies-outbreak areas and the model of its possible spread. We used a Cross-K function to investigate the spatial clustering between the locations of dog rabies cases and the feeding points of a stray dog feeder. We then observed the social interaction of dogs in a community using a metapopulation analysis and further simulated the possible spread of rabies within this population. We found that the reported rabies cases were spatially clustered with the routes of the dog feeder. Therefore, more sustainable stray dog management is required. Based on our community dog observations, we found 20 groups comprising 222 dogs with an average of 11 dogs per group. In our infectious model, we suggested that 47.7% of dogs are likely to be infected in a year if no interventions are implemented. Therefore, the veterinary authorities should rigorously strengthen their rabies prevention and control strategies to protect both animal and human health.

## Introduction

Rabies is a fatal zoonotic disease caused by rabies virus belonging to the genus *Lyssavirus* within the family *Rhabdoviridae*. Cases of this disease have been reported in 150 countries globally with 59,000 human deaths worldwide annually. Over half of these human cases are <15 years old (1). In animals, a wide variety of mammals serve as hosts for the virus, for example, dogs, cats, cattle, buffaloes, horses, weasels, bats, foxes, and raccoons (2). However, 95% of the cases in dogs and cats were reported in Asia and Africa where stray animals were present.

Thailand is one of the rabies endemic countries with its rapid spread across the country in recent years. In 2017, 87% of the rabies tested positive animal samples (1281/1469) were dogs (3). Thus, dogs are considered the main reservoir of rabies in Thailand. The occurrence of dog rabies cases has been continuously detected in all regions of Thailand mainly due to the failure of rabies vaccination coverage in the dog population from lack of accurate information on targeted population and socioeconomic factors of pet owners, lack of effective dog population management and control, and limited participation of local administration. Moreover, in 2012–2018, animal rabies cases had been reported in all provinces located in the western region of the country through both active and passive surveillance systems by the department of livestock development (DLD). However, the estimated distribution of dog population in the western region was less than that in other regions. Within this period, the highest number of cases was found in Prachuap Khiri Khan province, in which 85.7% of the cases (36/42) were dogs, while the rest were cats (5/42) and horses. In 2016–2018, most of the cases [30] were recognized in Pranburi district. This area is, hence, worth exploring extensively for rabies transmission in the dog population.

Mathematical models for infectious diseases use equations to direct the changes in the spreads. One of the most popular modeling structures is the compartmental model owing to its clarity and simplicity (4). The compartmental model divides a certain population into different levels of subpopulation according to the health status (5). The Susceptible–Exposed–Infectious–Recovered (SEIR) model is one of the typical examples separating individuals into four health compartments (6). The SEIR model was previously used to explain the spread of different diseases such as varicella (7) and rabies (8). However, the model assumes homogeneous mixing within the population, and in reality, individuals heterogeneously contact each other. A more precise model such as metapopulation was then proposed.

The metapopulation model divides the entire population into subgroups (9) to study the change and predict the distribution of these populations. The model has also been employed in epidemiological studies and was also used to study the spread of rabies and the efficiency of the vaccine in Tanzania (10). In Thailand, especially in rural communities, dogs from different households always roam around together in small groups. The metapopulation framework is hence suitable to model the spread of rabies virus within and among these groups of dogs.

The present study, therefore, aimed to simulate the spread of rabies virus among dogs gathering in different subpopulations and roaming around the rabies outbreak area. In addition, we analyzed the spatial clustering between the locations of reported dog rabies cases and the feeding points of a dog feeder.

## Materials and Methods

### Field Data Collection

**Figure 1:**
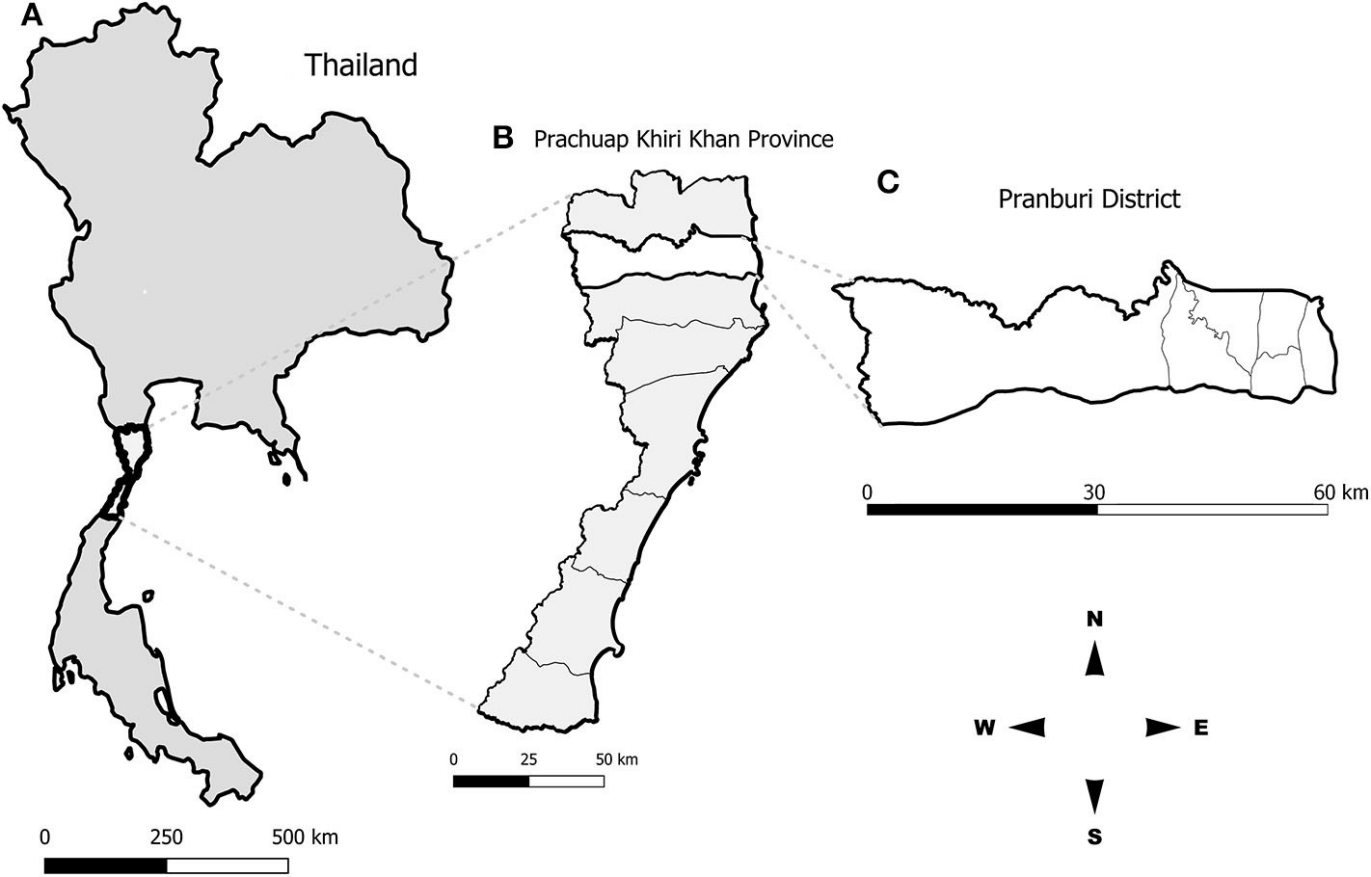
Study area in **(A)** Thailand, **(B)** Prachuap Khiri Khan province, **(C)** Pranburi district.

In January 2018, we went to Pranburi district, Prachuap Khiri Khan province (Figure 1), to retrospectively collect geographical locations of rabies cases in dogs within the district during 2016–2018 from the database of the DLD, Ministry of Agriculture and Cooperatives, Thailand. Most of the rabies cases in Pranburi district occurred in stray dogs, mainly fed by local feeder. A stray dog feeder was identified during free-roaming dog observations to indicate stray dog living areas. From our observations for four consecutive days and after interviewing local officials and residents, only a single stray dog feeder who was feeding stray dogs throughout the area daily for more than 20 years, was identified. Therefore, we tracked the movement of a dog feeder in the area to investigate the feeding routes of stray dogs. On the tracking day, we followed the local dog feeder all day long from 6 a.m. to 6 p.m. We recorded all the feeding routes and feeding points with the application “Google map” equipped in a mobile device. All recorded geographical coordinates were subsequently extracted and mapped using the QGIS program version 2.18.21 (https://www.qgis.org/).

**Figure 2:**
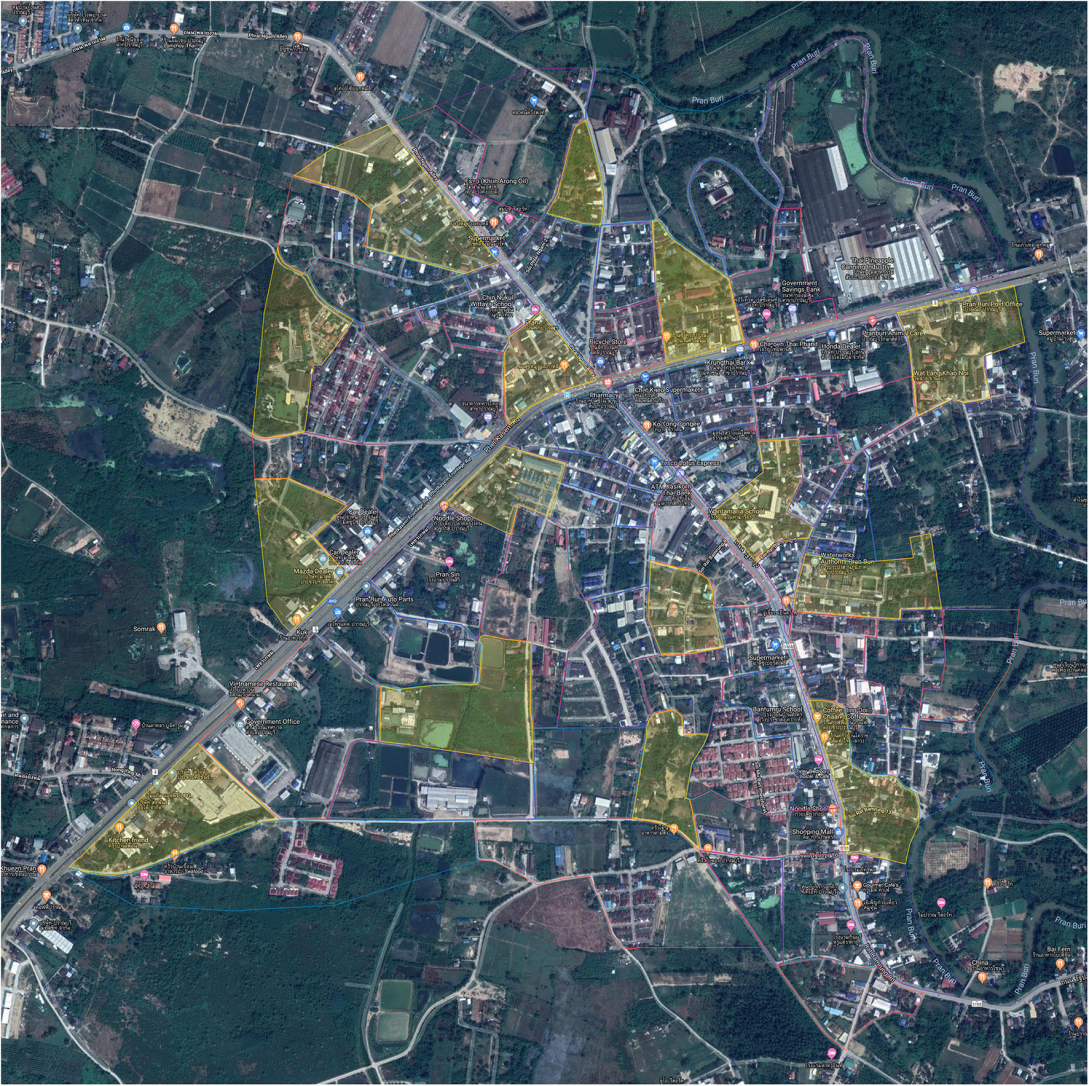
Fifteen sample blocks of Pranburi district central area conducting free roaming dog observation.

In addition, we observed a free-roaming dog population on eight consecutive occasions (from 6 a.m. to 8 a.m. and 4 p.m. to 6 p.m. for four consecutive days). According to the World Society for the Protection of Animals (WSPA) guidelines (11), a Google map of the Pranburi district central area was used to divide the area into 65 blocks (3.75 km^2^) with each block assigned one of the four colors (blue, yellow, red, and green). A total of 15 yellow blocks (0.75 km^2^) were randomly selected for the dog surveys (Figure 2). The distance between each block to the adjacent blocks ranged from 0.05 to 0.5 km. Five survey teams from two persons were formed. Each team was assigned three sample blocks and traveled through each block on the foot. Any dogs that were in public areas and were not currently confined while walking throughout the sample blocks were documented. The dog information such as the photographs, physical characteristics, gender, age, and GPS location were collected using a smartphone application, Epicollect5 (developed by Imperial College, London) (12) to individually identify each dog in every observed occasion.

### Spatial Analysis

Visual inspection of the study sites suggested that feeding points and dog rabies cases were spatially clustered. We hypothesized that these two groups of locations were spatially clustered. Cross-K function with the “Spatstat” statistical package (13) in program R version 3.6.3 (14) was used to analyze the spatial clustering between feeding points and dog rabies cases. To examine the spatial relationships between dog rabies points and feeding points, the null hypothesis was made that dog rabies points were distributed according to the complete spatial randomness (CSR), regardless of the distribution of feeding points. Statistical inference of the difference between the observed Cross-K function and the expected Cross-K function with the confidence envelopes at the 99.9% confidence level generated by the random labeling approach through Monte Carlo simulation with 999 permutations was examined.

### Infectious Metapopulation Modeling

**Figure 3:**
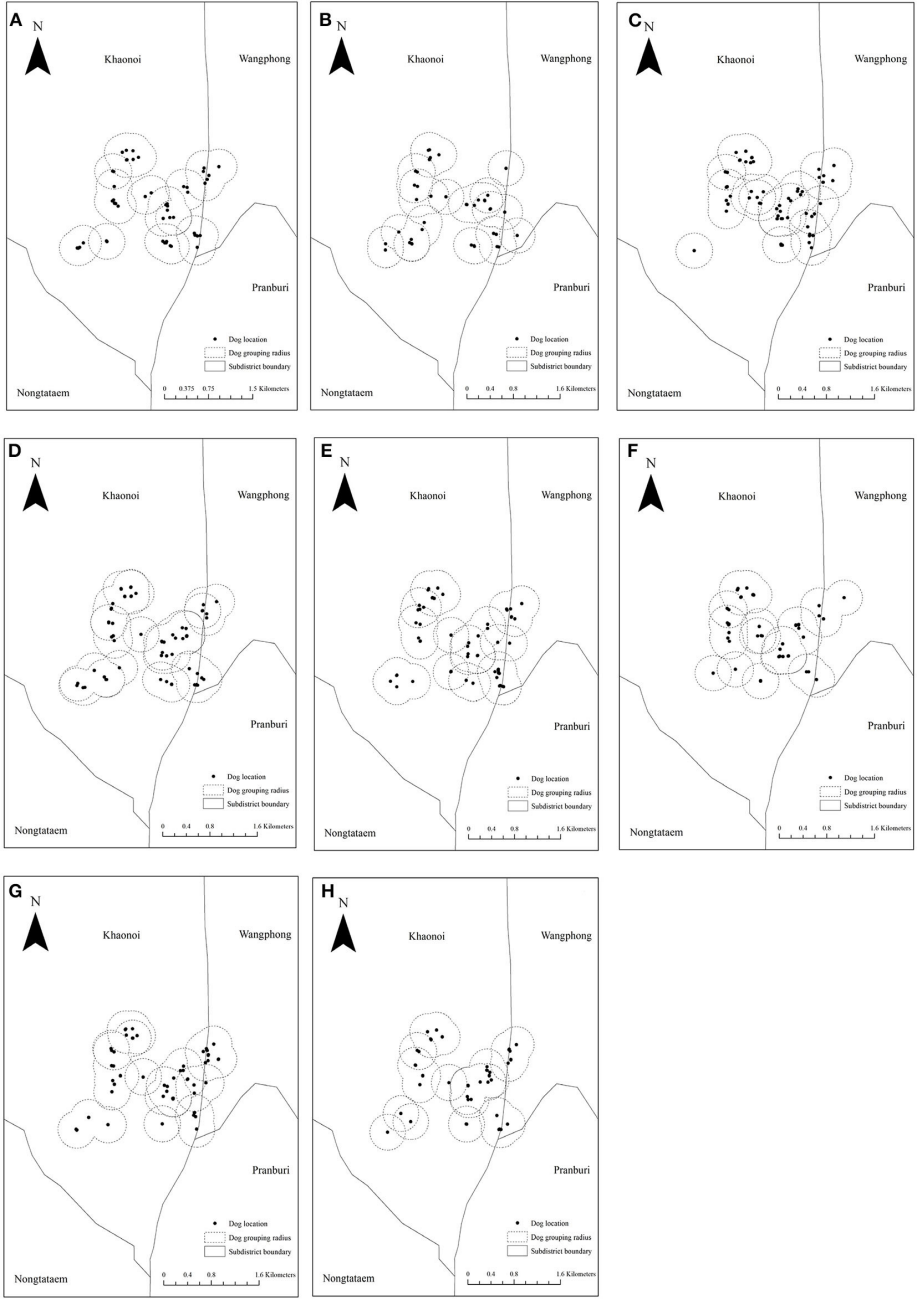
The division of the patches according to our dog observations on eight consecutive occasions (four morning and four evening). **(A–H)** The location of dogs on each observed period and the dotted circle represents the boundary of each patch.

**Figure 4:**
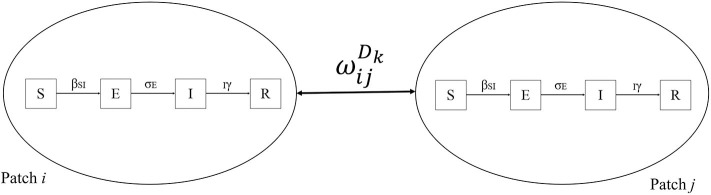
A conceptual framework of the infectious metapopulation model of rabies in dogs in Prachuap Khiri Khan province, Thailand. The symbol $W_{ij}^{D_{k}}$ represents the dog movement across patches *i* and *j* on day *k*. The S, E, I, and R denote Susceptible, Exposed, Infectious and Recovered compartments. The parameters β, σ, and γ represent transmission rate, latency rate, and death rate, respectively.

Based on our dog observations over four consecutive days, we collected the GPS locations of each individual dog on eight occasions. We found that dogs generally gathered within a radius of 300 m (Figure 3). As dogs gathered in different small groups, we accordingly subdivided the observed dogs into patches. Subsequently, we counted the number of dogs moving across the patches over the observed period. To simulate the between-patch movements, we used the actual number of dogs moved across patches observed over a 4-day period and then repeated those until we reached one year (365 days). The dog movement was governed by the following equations:

$$\begin{array}{r} W_{ij}^{D_{k}}=M_{ij}^{D_{o}}\;,\;o=\;r_{k}, \end{array}$$

$$\begin{array}{r} r_{k}=\;\left\{ \begin{array}{c} k,\text{ if }k<5\\ Rem\left(\frac{k}{4}\right),\text{ if }k\;\geq 5\\ 4,\text{ if }Rem\left(\frac{k}{4}\right)=0 \end{array}\right. \end{array}$$

where $W_{ij}^{D_{k}}$ is the number of dogs moved from patch *i* to patch *j* on day *k*. $M_{ij}^{D_{0}}$ represents the observed number of dogs moved across patches observed on day 0 in a 4-day period. The *r_k_* notes the remainder (*Rem*) of the simulated day (*k*) divided by 4 to repeat what was observed to complete the modeled period of 1 year. A metapopulational SEIR model was then constructed to simulate the spread of rabies as conceptualized in Figure 4.

The SEIR model was simulated using ordinary differential equations as follows:

$$\begin{array}{l} \frac{dS}{dt}=\;-\beta SI\\ \frac{dE}{dt}=\;\beta SI-\sigma E\\ \frac{dI}{dt}=\;\sigma E-I\gamma \\ \frac{dR}{dt}=\;I\gamma \\ N=S+E+I+R \end{array}$$

where S, E, I, and R refer to susceptible, exposed, infectious, and recovered compartments, respectively. The parameters β, σ, and γ, are the transmission rate, latency rate, and death rate, respectively

We assigned the infectious status of each patch based on the history of dog rabies occurrence in 2016–2018. The patches with previous rabies notification would be infected first. In our model, dogs moving across patches were randomly selected from all compartments. Based on our observation, dogs always moved back to their original locations after roaming. Hence, we modeled it accordingly. The parameters governing the rabies transmission dynamics are shown in Table 1. We used the package “SimInf” (19) in program R, and the model was run for 365 days.

**Table 1:** Parameters used in the SEIR model of dog rabies transmission.

**Parameter**	**Value**	**References**
Basic reproductive number (R_0_)	2.44	Kitala et al. (15)
Latency rate (σ)	0.034	Laager et al. (16)
Death rate (γ)	0.32	Hampson et al. (17)
Transmission rate (β)	0.78	Calculated from R${}_{0}^{*}\gamma$ (18)

## Results

### Field Observations and Spatial Analysis

**Figure 5:**
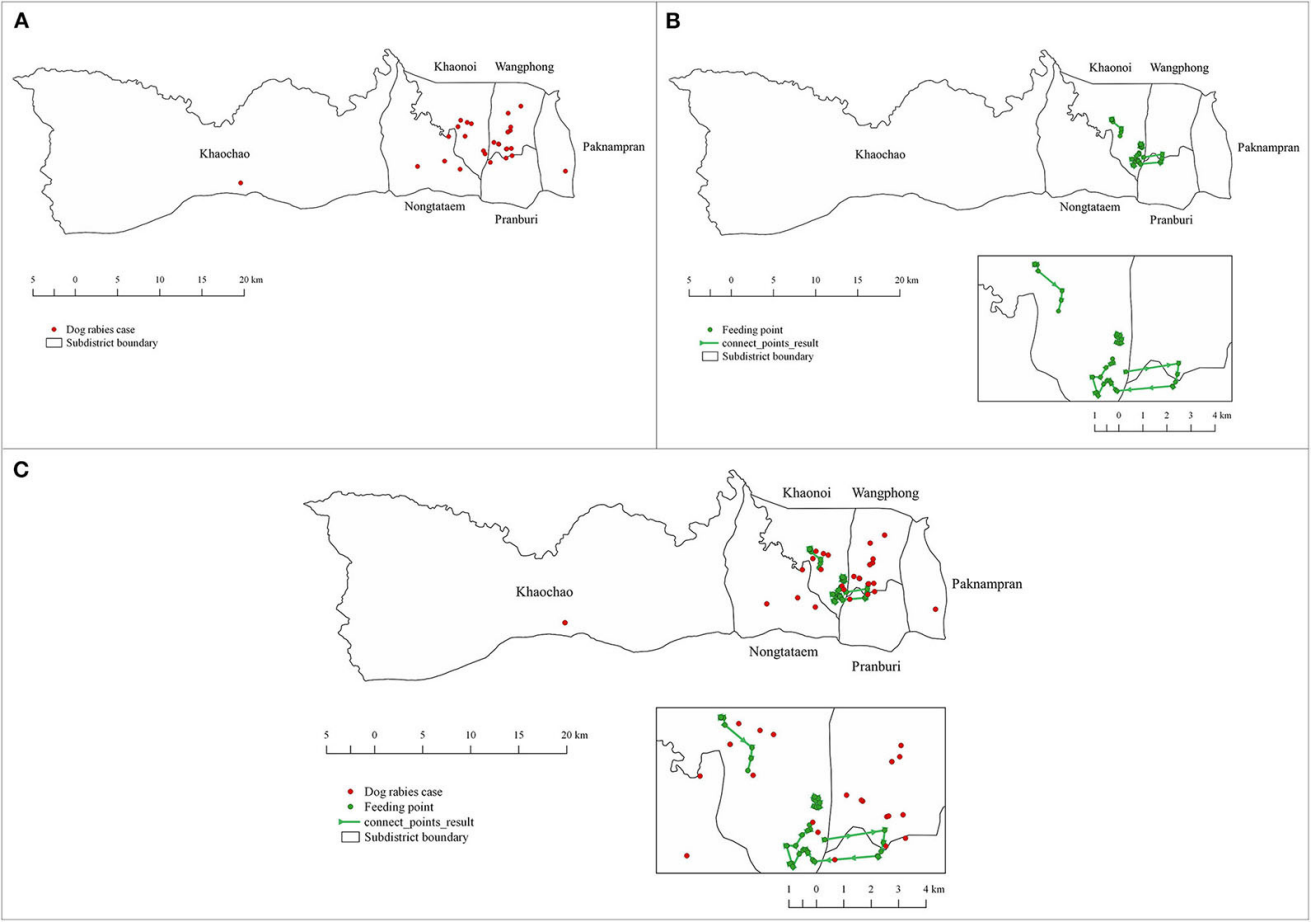
Locations of rabies cases in dogs and the routes of a dog feeder in Pranburi district, Prachuap Khiri Khan province, Thailand. **(A)** Locations of dog rabies cases during 2016–2018 (red dots). **(B)** The routes of a dog feeder (green line) with feeding points (green dots). **(C)** Combined map of rabies case locations and the routes of a dog feeder.

**Figure 6:**
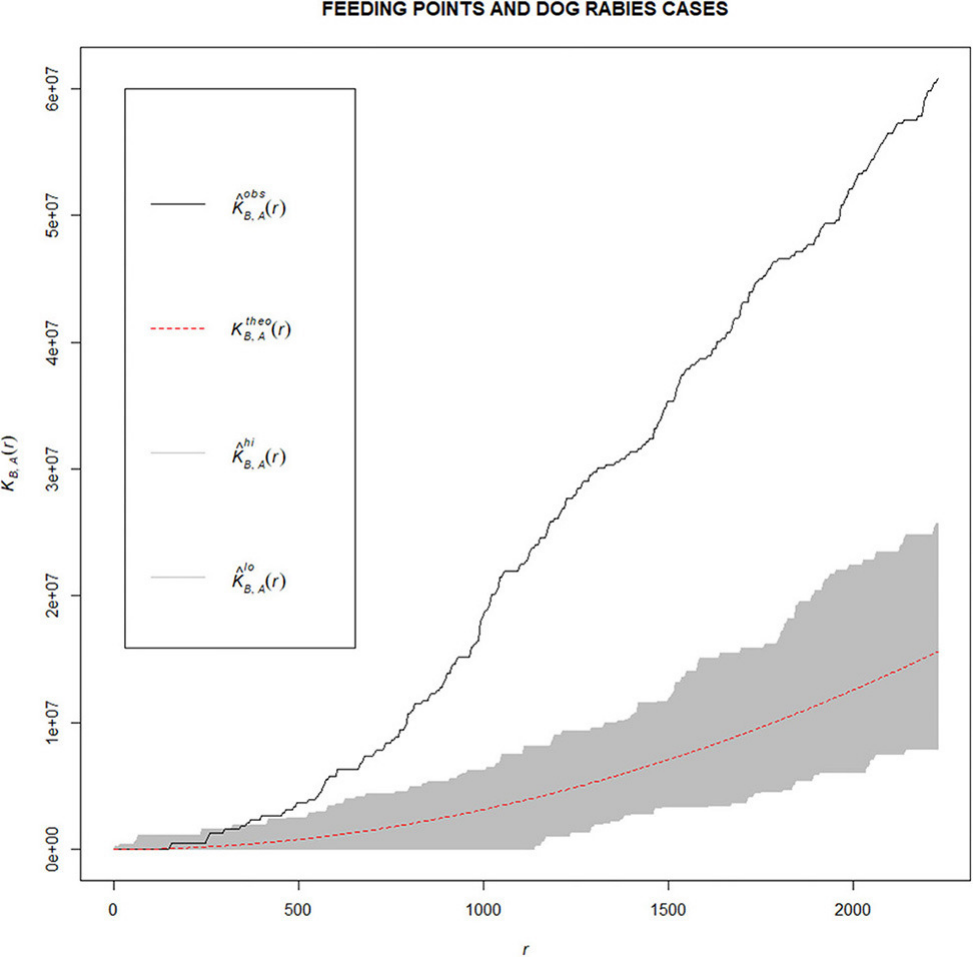
Observed K–function (black line), theoretical K–function (red dashed line) and confidence envelopes (gray area) for dog rabies cases (denoted as A) and feeding points (denoted as B). (K^obs^: observed K–function, K^theo^: theoretical K–function, K^hi^: upper envelope, K^lo^: lower envelope).

From the DLD database, we found 30 dog rabies cases in 2016–2018 in the district (Figure 5A). Of these, 15, 7, and 8 cases were recorded in those consecutive years, respectively. In our stray dog feeder tracking, we found that the feeder traveled to feed roughly 169 stray dogs from 7.30 am to 5 pm once a day covering a distance of 30 km, making a total of 40 stops along three different routes (Figure 5B). After overlapping the locations of dog rabies cases and the routes of dog feeder (Figure 5C), we suspected that these two groups of locations were spatially clustered. Our hypothesis was confirmed with the Cross-K function that the relative spatial clustering pattern was observed. Figure 6 shows that the observed K–function lies above the upper envelope curve in the range of 350–2230 m at the 0.001 significance level under the CSR hypothesis, indicating that the dog rabies cases spatially clustered around feeding points within this estimated range. This indicates that dog rabies case points prefer to cluster around feeding points or dog rabies cases are attracted to feeding points.

**Figure 7:**
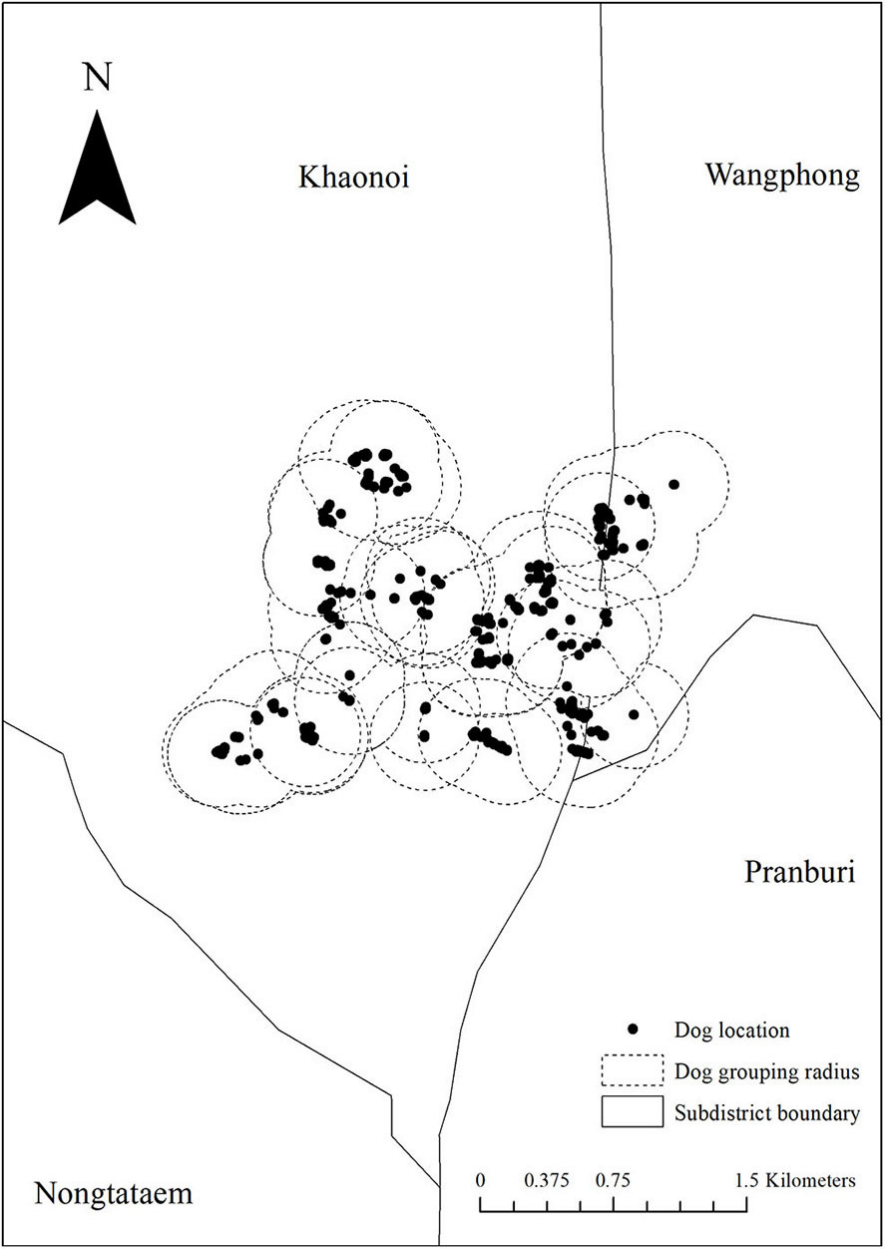
Metapopulation of 20 dog groups during the observed period.

In our dog observations on eight consecutive occasions, we could identify 222 individual dogs (detailed in Supplementary Appendices I, II). Based on the cutoff patch radius of 300 m (Figure 3), these dogs were then subdivided into 20 patches as shown in Figure 7. The number of dogs in each patch is shown in Table 2. Each patch contained 3–33 dogs (median = 8), and the movement of dogs across patches is summarized in Table 3. We observed that 1–9 dogs (median = 3) moved across patches in each movement. According to the DLD data, dog rabies was notified from 5 out of 20 patches with 1–2 dogs. We then initiated the infection in these five patches.

**Table 2:** Number of dogs in each patch.

	**Patch**																			
	**1**	**2**	**3**	**4**	**5**	**6**	**7**	**8**	**9**	**10**	**11**	**12**	**13**	**14**	**15**	**16**	**17**	**18**	**19**	**20**
Number of dogs	30	13	13	25	6	33	18	10	2	11	3	3	2	6	3	11	4	6	20	3

**Table 3:** The movement of dogs across patches during the observed period (4 days).

**Day**	**Patch of origin**	**Patch of destination**	**Number of dog moved**
1	1	2	1
	1	14	4
	2	1	3
	6	7	1
	7	6	3
	7	17	5
	17	18	2
	2	1	2
	6	7	3
	7	6	9
	14	15	4
2	1	2	6
	2	1	6
	2	3	2
	3	2	7
	4	5	5
	6	7	7
	7	6	8
	8	10	4
	15	16	2
	16	17	3
	1	2	1
	2	1	3
	2	3	2
	6	7	2
	6	19	6
	7	6	3
	11	16	3
	18	19	1
3	1	2	1
	2	1	3
	2	3	2
	6	7	1
	7	6	4
	2	1	2
	3	2	5
	6	7	2
	6	18	7
	6	19	6
	7	6	5
	18	19	2
4	1	3	1
	2	1	2
	2	3	2
	6	7	3
	7	6	6
	8	10	1
	2	1	2
	2	3	2
	7	6	3

### Rabies Transmission Modeling Results

**Figure 8:**
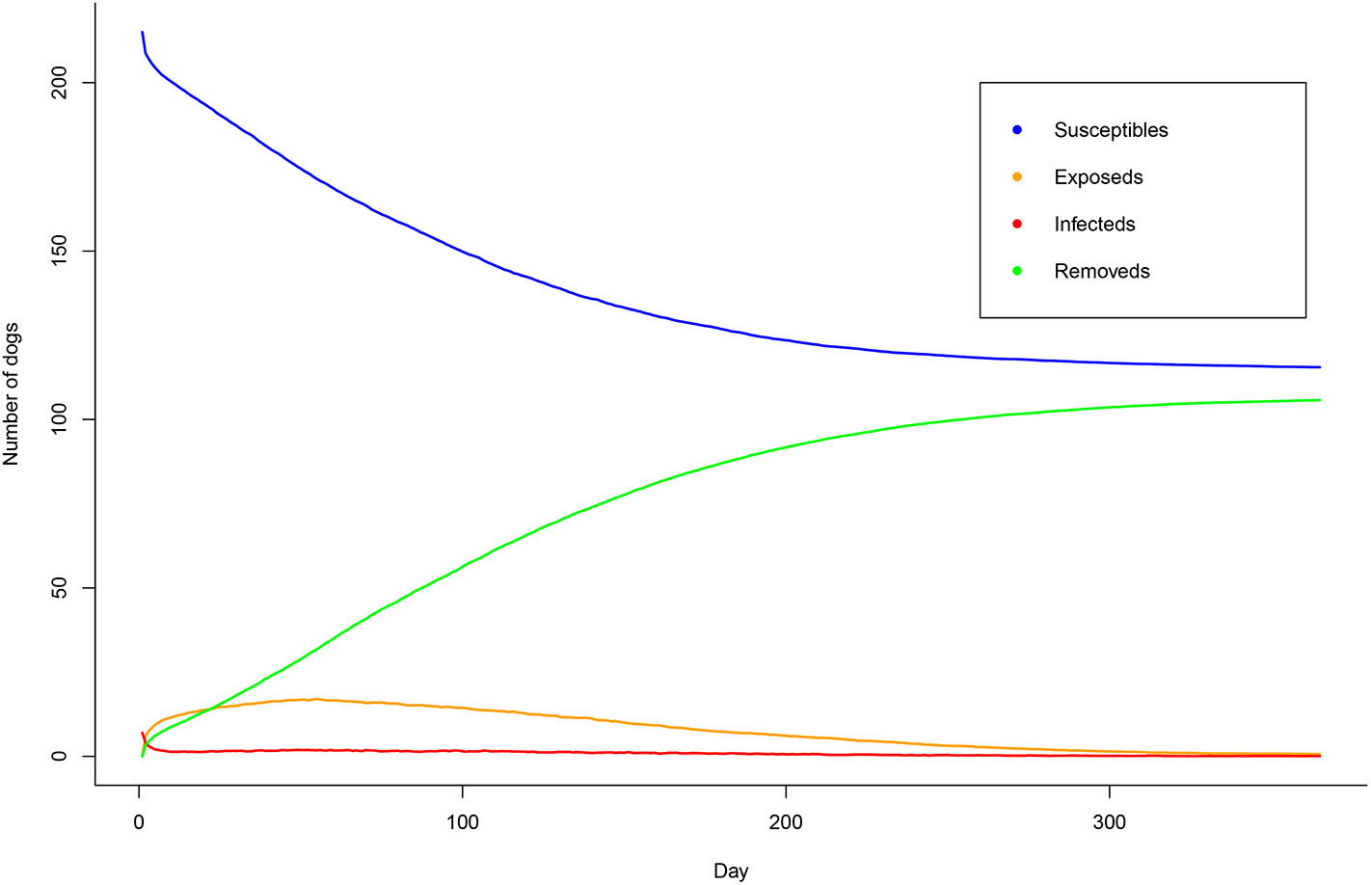
The spread of rabies in dog populations based on a metapopulational SEIR model.

We observed that some patches had only a few dogs with a minimum number of 2 dogs (Table 2). Besides, our results were based on non-homogenous mixing populations. The overall changes over time is hence depicted in Figure 8 to show the likely spread of rabies in these dog populations. Based on our results, we found that the dogs were gradually infected. Within 1 year, 47.7% of dogs (106/222) were infected and died if there was no intervention.

## Discussion

The present study compared the previous rabies cases in dogs with the routes of a dog feeder to initially investigate the spatial association of the stray dog feeding behavior and the occurrences of rabies cases in the area. We then observed the formation of social interaction by dogs in a community using a metapopulation analysis and further simulated the likely spread of rabies within this population.

We found that most rabies cases in dogs were notified in the urban areas of the study district (Figure 5A). These areas are likely well-inhabitable for stray and owned free-roaming dogs, as different large markets are present to serve people living in these big communities. Concurrently, these markets act as feeding sources for unvaccinated stray dogs. Once the virus is introduced, it may easily spread among the dog population (10). It was evident in a previous study that the two strategies can effectively mitigate the spread of rabies virus, especially in stray dog populations (20).

Interestingly, a dog feeder could travel over a distance of 30 km daily (Figure 5B). As per our observations, this feeder had fed more than 169 dogs along the routes. This stray dog feeding behavior may have contributed to the increase in the stray dog population. However, dog population density is not the only risk factor for the spread of rabies (21, 22). There are several factors worth exploring in future studies, such as vaccination coverage, human and dog behaviors, etc. We also observed that the dog feeder was familiar with all dogs in the feeding routes. This is beneficial for the dog vaccination program as the dog feeder can restrain these stray dogs. However, fully blaming these feeders is not convincing. More sustainable ways of stray dog control, such as shelter establishment, public education as well as law enforcement on dog abandon punishment, should be implemented (20).

Based on our spatial analysis, we found spatial clustering between the feeding routes and the locations of dog rabies cases (Figure 6). The relatively high density of stray dogs is probably an important factor in the incidence of dog rabies. Therefore, the area with high density of stray dogs can be targeted for surveillance activities. A risk-based surveillance program should be developed to increase the sensitivity of the surveillance. In addition, the measures aiming to reduce stray dog population density must be used to control rabies. However, we only studied a certain area with only a single observed dog feeder. To generalize this finding, a future study expanding over a larger area in different regions of the country is suggested. In addition, several dog feeders should be included to better explore dog feeding behaviors, which may vary due to different factors and conditions.

In the metapopulation, we observed that dogs moved substantially across patches (Table 3 and Figure 7). Beyer et al. suggested that the virus is likely to spread across villages infecting different dog populations depending on the size of the population and the distance between villages (10). It was also found that the introduction of new dogs into the population may increase the risk of disease transmission (16). However, we observed only 222 dogs within a certain community in this study. An extended study is recommended to include other factors, for instance, distances between patches and new dog importation. In addition, we manually observed the dogs over four consecutive days, which was labor-intensive and is not a sustainable practice in the long run. A future study using automatic machine learning technology to identify and record dogs is suggested. This approach is also applicable to dog census database registration. Nonetheless, the development of such technology requires a multidisciplinary approach and time.

We acknowledge some potential limitations that we faced in this study. First, we manually observed the dog movements only in a 4-day period and modeled the movements of the dogs within and across the patches accordingly. In a future study, we suggest using advanced tools such as video cameras or GPS trackers to continue observing the movements over a longer period of time. Thus, the dog movement data would be more accurate and elaborate. Second, we developed a baseline infectious model in which no interventions were assigned. However, once a rabies outbreak occurs, control measures must be implemented immediately. In this study, we mainly illustrated the impact of rabies spread without any interventions to raise awareness among policymakers and the general public on this deadly zoonosis. In a future study, basic rabies control strategies such as vaccination, neutralization, depopulation, and mixed interventions should be developed based upon this baseline model to determine the best strategy for Thailand.

Based on our modeling results, we found that half of the dogs would be infected if no interventions were implemented (Figure 8). It was noteworthy that we modeled under the worse-case scenario where R_0_ was set at 2.44 (15). This value is relatively high for rabies transmission compared to other studies. In general, R_0_ of rabies spread is <2 (23). In this study, we intended to raise awareness among the public and all sectors involved in planning rabies prevention and control programs. Nonetheless, our results were still in line with a previous study that suggested that approximately two fifth of the dog population would die within 6 months in an uncontrolled situation (24). Our modeling structure is relatively flexible and ready to be used as a baseline model for interventional modeling; for example, models of how vaccination programs or dog neutralization can help mitigate the outbreak. Indeed, Thai DLD have been consecutively implementing different control measures for rabies virus in dogs including those aforementioned strategies such as annual nationwide dog vaccination campaigns and dog population control programs (free services of dog neutralization for owned and stray dogs). In the future study, data on dog population dynamics is needed. The interventional model would then include these factors to make it more responsive to the changes of populations.

In conclusion, we found spatial clustering between reported cases of rabies in dogs and the feeder routes. A more sustainable stray dog management, such as shelter establishment, is required. An uncontrolled rabies outbreak may affect half of the dog population and pose a great risk to people living closely with these dogs. The veterinary authorities should strengthen their rabies prevention and control strategies to protect both animal and human health.

## Data Availability Statement

The original contributions presented in the study are included in the article/Supplementary Materials, further inquiries can be directed to the corresponding author/s.

## Author Contributions

PK, SS, and PJ contributed to collection and primary analysis of data. AW and KL contributed to the concept, design, data analysis and interpretation, and the preparation of the manuscript. All authors contributed to the writing of, reviewed, and approved the contents of this manuscript.

## Conflict of Interest

The authors declare that the research was conducted in the absence of any commercial or financial relationships that could be construed as a potential conflict of interest.
