# Supplementary material for: The Spread of Rabies Among Dogs in Pranburi District, Thailand: A Metapopulation Modeling Approach
Source: Front Vet Sci. 2020 Nov 19;7:570504. doi: 10.3389/fvets.2020.570504 (PMC7710610; doi:10.3389/fvets.2020.570504)
Supplement: Supplementary file 1 [file Data_Sheet_1.PDF]

Appendix l

Signalment of dogs observed in the study

Polygon	ID	Sex	Age	physical characteristic		
				Color	Hair	Body
P11	1	unknown	adult	brown	long	overweight
P11	2	male	adult	light brown	short	overweight
P11	3	female	adult	light brown	short	normal
P11	4	male	adult	light brown	long	normal
P11	5	female	adult	light brown	short	overweight
P11	6	male	adult	white	short	normal
P11	7	male	adult	black	long	normal
P11	8	male	adult	white and black	long	overweight
P11	9	male	adult	light brown	long	normal
P11	10	female	pediatric	black	short	normal
P11	11	male	pediatric	brown	short	normal
P11	12	female	adult	light brown	long	normal
P12	1	female	adult	light brown	long	underweight
P12	2	female	pediatric	light brown and white	short	underweight
P12	3	female	adult	white and black	long	normal
P12	4	male	pediatric	white	short	normal
P12	5	male	adult	dark brown	long	underweight
P12	6	male	adult	black	short	overweight
P12	7	male	adult	cream	long	overweight
P12	8	unknown	adult	cream	short	normal
P12	9	male	adult	brown	short	normal
P12	10	unknown	adult	light brown	short	normal
P12	11	unknown	adult	brown	short	normal
P12	12	male	adult	white and brown	short	overweight

Appendix l *(Continued)*

Polygon	ID	Sex	Age	physical characteristic		
				Color	Hair	Body
P12	13	male	adult	brown and black	short	normal
P12	14	male	adult	black	short	normal
P12	15	female	adult	white and light brown	long	normal
P12	16	male	adult	white	long	normal
P12	17	male	adult	brown	long	normal
P12	18	male	adult	black	short	normal
P13	1	female	adult	black	long	overweight
P13	2	male	adult	brown and black	long	normal
P13	3	female	adult	light brown	short	normal
P13	4	female	adult	black	short	normal
P13	5	female	adult	light brown	short	normal
P13	6	male	adult	white	short	normal
P13	7	male	adult	black and white	long	underweight
P13	8	male	adult	light brown	long	normal
P13	9	male	adult	white and black	short	normal
P13	10	male	adult	light brown and white	long	normal
P13	11	female	adult	Mixed	short	underweight
P13	12	male	adult	light brown	long	normal
P13	13	female	adult	brown	long	normal
P13	14	male	adult	light brown	long	normal
P13	15	female	adult	light brown	long	normal
P13	16	female	adult	brown	short	normal
P13	17	male	adult	black	short	normal
P13	18	female	adult	black and white	long	normal

Appendix l *(Continued)*

Polygon	ID	Sex	Age	physical characteristic		
				Color	Hair	Body
P21	1	male	adult	black and brown	short	normal
P21	2	female	adult	light brown	short	normal
P21	3	female	adult	brown	short	overweight
P21	4	female	adult	black	unknown	underweight
P21	5	female	adult	black	short	underweight
P21	6	male	adult	brown and white	short	overweight
P21	7	male	adult	black	short	normal
P21	8	male	adult	black	unknown	normal
P21	9	unknown	adult	black	unknown	normal
P21	10	unknown	adult	black	unknown	underweight
P21	11	female	adult	brown	unknown	underweight
P21	12	unknown	adult	brown	unknown	normal
P21	13	unknown	adult	white and black	unknown	overweight
P21	14	unknown	adult	white and grey	unknown	overweight
P21	15	unknown	adult	grey	unknown	overweight
P21	16	unknown	adult	black	unknown	overweight
P21	17	unknown	adult	black	unknown	overweight
P21	18	unknown	adult	black	unknown	normal
P21	19	unknown	adult	black	unknown	underweight
P21	20	unknown	adult	black and white	unknown	overweight
P21	21	unknown	adult	black and white	unknown	normal
P21	22	unknown	adult	black and white	unknown	overweight
P21	23	female	pediatric	black and white	short	normal
P21	24	unknown	adult	black	short	normal

Appendix l *(Continued)*

Polygon	ID	Sex	Age	physical characteristic		
				Color	Hair	Body
P21	25	unknown	adult	black	short	normal
P21	26	unknown	adult	black	short	normal
P21	27	female	adult	brown and white	unknown	normal
P21	28	unknown	adult	Mixed	unknown	normal
P21	29	unknown	adult	Mixed	unknown	normal
P21	30	female	pediatric	black and white	short	normal
P21	31	male	adult	brown and white	short	normal
P22	1	male	adult	brown and white	long	normal
P22	2	unknown	adult	brown and white	long	normal
P22	3	male	adult	black and brown	unknown	normal
P22	4	female	adult	black and brown	unknown	normal
P22	5	male	adult	brown	unknown	normal
P22	6	male	adult	Mixed	unknown	normal
P22	7	male	adult	white	unknown	normal
P22	8	male	adult	black	unknown	overweight
P22	9	male	adult	brown and white	short	normal
P22	10	male	adult	brown	short	normal
P22	11	male	adult	white and black	short	normal
P22	12	female	adult	brown and black	short	normal
P22	13	male	adult	brown	unknown	normal
P22	14	female	adult	white	unknown	overweight
P22	15	male	adult	brown and white	unknown	normal
P22	16	male	adult	white and brown	long	normal
P22	17	female	adult	brown and white	unknown	overweight

Appendix l *(Continued)*

Polygon	ID	Sex	Age	physical characteristic		
				Color	Hair	Body
P22	18	female	adult	black and white	unknown	normal
P22	19	male	adult	white	unknown	overweight
P22	20	male	adult	brown	long	normal
P22	21	female	adult	black	short	normal
P22	22	male	adult	brown and grey	short	normal
P23	1	male	adult	white and brown	unknown	normal
P23	2	female	adult	brown	short	overweight
P23	3	female	adult	dark brown	short	overweight
P23	4	male	adult	white and brown	long	normal
P23	5	male	adult	black	short	normal
P23	6	male	adult	brown	short	normal
P23	7	female	adult	brown	long	underweight
P23	8	female	adult	black	short	normal
P23	9	unknown	adult	white and black	long	normal
P23	10	unknown	adult	white	short	normal
P23	11	unknown	adult	brown	long	normal
P23	12	unknown	adult	black and white	long	overweight
P23	13	female	adult	brown and white	short	underweight
P23	14	male	adult	brown	short	normal
P23	15	female	adult	black	unknown	underweight
P23	16	male	adult	brown	short	normal
P23	17	male	adult	dark grey	short	normal
P23	18	female	adult	black	short	underweight
P23	19	female	adult	brown and white	unknown	normal

Appendix l *(Continued)*

Polygon	ID	Sex	Age	physical characteristic		
				Color	Hair	Body
P23	20	female	pediatric	grey	short	normal
P23	21	female	adult	grey	short	normal
P23	22	male	adult	white and brown	short	normal
P23	23	female	adult	grey	long	normal
P23	24	male	adult	black	unknown	normal
P23	25	unknown	pediatric	black	unknown	normal
P23	26	female	adult	brown	short	normal
P23	27	male	adult	grey	short	normal
P23	28	unknown	adult	black	unknown	normal
P23	29	unknown	adult	dark brown	unknown	overweight
P23	30	unknown	adult	light brown	unknown	normal
P31	1	female	adult	cream	long	normal
P31	2	female	adult	light brown	long	normal
P31	3	female	adult	dark grey	long	normal
P31	4	male	adult	brown and white	short	normal
P31	5	unknown	pediatric	brown	short	underweight
P31	6	female	pediatric	brown and black	short	underweight
P31	7	female	adult	cream	short	normal
P31	8	female	adult	white	long	normal
P31	9	female	adult	brown	long	normal
P31	10	male	adult	cream	short	normal
P31	11	male	adult	red	short	normal
P31	12	female	adult	Mixed	short	normal
P31	13	unknown	adult	brown	short	underweight

Appendix l *(Continued)*

Polygon	ID	Sex	Age	physical characteristic		
				Color	Hair	Body
P31	14	female	adult	black	short	normal
P31	15	male	adult	black	short	normal
P31	16	female	adult	white and black	short	normal
P31	17	unknown	adult	red	short	underweight
P31	18	unknown	adult	white	long	normal
P31	19	female	adult	white	short	normal
P31	20	male	adult	white	short	normal
P31	21	unknown	adult	cream	short	normal
P31	22	unknown	adult	black and grey	short	normal
P31	23	female	adult	brown	short	normal
P31	24	unknown	adult	white	short	normal
P31	25	male	adult	cream	short	overweight
P31	26	female	adult	brown	short	normal
P32	1	male	adult	black	short	normal
P32	2	male	adult	red	short	normal
P32	3	male	adult	red	short	normal
P32	4	male	adult	black and brown	short	normal
P32	5	female	adult	brown	short	normal
P32	6	female	adult	black	short	normal
P32	7	male	adult	black	short	normal
P32	8	male	adult	black	short	normal
P33	1	female	adult	white and black	long	normal
P33	2	female	adult	light brown	short	normal
P33	3	male	adult	grey	short	normal

Appendix l *(Continued)*

Polygon	ID	Sex	Age	physical characteristic		
				Color	Hair	Body
P33	4	male	adult	black	long	normal
P33	5	unknown	adult	white	long	normal
P33	6	female	adult	black	short	normal
P33	7	female	pediatric	black	short	normal
P33	8	female	pediatric	black	short	normal
P33	9	unknown	pediatric	brown	short	underweight
P33	10	unknown	adult	white and black	long	normal
P33	11	unknown	adult	white	short	overweight
P33	12	unknown	adult	black	long	normal
P33	13	unknown	adult	brown	short	normal
P33	14	male	adult	red	short	overweight
P33	15	unknown	adult	white	long	normal
P33	16	female	adult	white and black	short	normal
P33	17	female	adult	white	long	normal
P41	1	male	adult	black	short	normal
P41	2	male	adult	gold	short	normal
P41	3	male	adult	black	short	normal
P41	4	male	adult	white	short	normal
P41	5	male	adult	white	long	normal
P41	6	female	adult	white	short	overweight
P41	7	male	adult	black	short	normal
P41	8	female	adult	gold	short	normal
P41	9	male	adult	brown	short	normal
P41	10	female	adult	black	short	normal

Appendix l *(Continued)*

Polygon	ID	Sex	Age	physical characteristic		
				Color	Hair	Body
P41	11	female	adult	brown	short	normal
P42	1	male	adult	brown	short	normal
P42	2	female	adult	black	short	normal
P42	3	unknown	adult	white	long	normal
P42	4	male	adult	cream	long	normal
P42	5	male	adult	brown	long	normal
P42	6	female	adult	dark grey	short	normal
P42	7	female	adult	gold	short	normal
P42	8	female	adult	white	short	normal
P42	9	male	adult	brown	short	normal
P43	1	female	adult	white	long	normal
P43	2	female	adult	brown	short	normal
P43	3	male	adult	Mixed	short	normal
P43	4	male	adult	grey	short	normal
P43	5	female	adult	white	short	normal
P43	6	male	adult	white	long	normal
P43	7	male	adult	white	long	normal
P43	8	male	adult	brown	long	normal
P43	9	female	adult	white	long	normal
P43	10	female	adult	brown	long	normal
P43	11	male	adult	black	short	normal
P43	12	male	adult	black	short	normal
P43	13	male	adult	black	short	normal
P43	14	male	adult	white	short	normal

Appendix l *(Continued)*

Polygon	ID	Sex	Age	physical characteristic		
				Color	Hair	Body
P43	15	male	adult	black	long	normal
P43	16	male	adult	grey	short	normal
P43	17	male	adult	white	long	normal
P43	18	unknown	adult	white	long	normal
P43	19	female	adult	gold	short	normal
P43	20	male	adult	white	long	normal
