# Supplementary material for: The Spread of Rabies Among Dogs in Pranburi District, Thailand: A Metapopulation Modeling Approach
Source: Front Vet Sci. 2020 Nov 19;7:570504. doi: 10.3389/fvets.2020.570504 (PMC7710610; doi:10.3389/fvets.2020.570504)
Supplement: Supplementary file 2 [file Data_Sheet_2.PDF]

Appendix ll

Location of dogs observed in the study

Polygon	ID	Location (latitude, longitude)							
		1	2	3	4	5	6	7	8
P11	1	(12.384351, 99.894754)	(12.38438, 99.89476)	(12.384342, 99.894767)	(12.38433, 99.89475)	(12.38432, 99.89471)	(12.38435, 99.89478)	(12.384324, 99.894826)	
P11	2	(12.384191, 99.894566)							(12.38507, 99.89553)
P11	3	(12.383855, 99.894877)		(12.383755, 99.895008)				(12.383771, 99.895065)	(12.38507, 99.89553)
P11	4	(12.383863, 99.894906)		(12.383809, 99.895)				(12.383771, 99.895072)	
P11	5	(12.383792, 99.895068)		(12.383751, 99.894996)	(12.38378, 99.89515)	(12.38384, 99.89498)	(12.38384, 99.89501)	(12.383752, 99.895076)	(12.38375, 99.89508)
P11	6	(12.383402, 99.894608)			(12.38451, 99.89501)				
P11	7			(12.385011, 99.895339)					
P11	8			(12.382672, 99.894754)				(12.38263, 99.894715)	
P11	9	(12.383402, 99.895457)				(12.383812, 99.894954)			
P11	10							(12.385, 99.896)	
P11	11							(12.385, 99.896)	
P11	12						(12.38517, 99.89496)		
P12	1		(12.388776, 99.894605)						

Appendix ll *(Continued)*

Polygon	ID	Location (latitude, longitude)							
		1	2	3	4	5	6	7	8
P12	2	(12.388804, 99.894608)	(12.38877, 99.8946)	(12.388763, 99.894614)		(12.38861, 99.89464)		(12.38877, 99.894649)	
P12	3	(12.388735, 99.894778)	(12.38865, 99.89489)	(12.388677, 99.894894)	(12.38876, 99.89473)	(12.38871, 99.89491)	(12.38864, 99.89493)	(12.388566, 99.895019)	(12.38868, 99.89493)
P12	4	(12.386424, 99.894844)		(12.386396, 99.894759)					
P12	5	(12.386438, 99.894849)	(12.38642, 99.89491)	(12.386474, 99.894877)	(12.38644, 99.8949)	(12.38642, 99.89496)	(12.38643, 99.89495)	(12.386441, 99.894941)	
P12	6				(12.38948, 99.89492)	(12.389021, 99.895509)			
P12	7				(12.38865, 99.89468)	(12.389298, 99.894828)	(12.38883, 99.89467)		(12.38885, 99.89465)
P12	8				(12.38663, 99.89431)				
P12	9				(12.38663, 99.89431)	(12.386649, 99.894316)			(12.38661, 99.89441)
P12	10				(12.38663, 99.89431)	(12.386649, 99.894316)			
P12	11				(12.38663, 99.89431)				
P12	12						(12.38661, 99.8947)		
P12	13						(12.38645, 99.89491)		
P12	14						(12.3864, 99.89489)		

Appendix ll *(Continued)*

Polygon	ID	Location (latitude, longitude)							
		1	2	3	4	5	6	7	8
P12	15							(12.389064, 99.89457)	
P12	16							(12.386485, 99.89465)	
P12	17							(12.386485, 99.89465)	
P12	18								(12.38663, 99.89437)
P13	1	(12.39061, 99.897751)		(12.39033, 99.897661)		(12.390389, 99.897155)			
P13	2	(12.390624, 99.897755)		(12.390179, 99.898379)		(12.39038, 99.897158)			
P13	3	(12.390578, 99.896803)	(12.39087, 99.89688)	(12.390548, 99.89674)	(12.39057, 99.89676)	(12.39047, 99.89686)	(12.39061, 99.89677)	(12.391066, 99.89687)	(12.39047, 99.89683)
P13	4	(12.390553, 99.896723)	(12.39073, 99.89683)	(12.390537, 99.896734)	(12.39061, 99.89676)	(12.39048, 99.89691)	(12.39054, 99.89674)		(12.39059, 99.89676)
P13	5	(12.390559, 99.896745)	(12.39056, 99.89674)	(12.390607, 99.896798)	(12.3906, 99.89676)		(12.39059, 99.89676)		
P13	6	(12.39172, 99.896095)	(12.39199, 99.89659)		(12.39179, 99.8962)	(12.39165, 99.8962)	(12.39191, 99.89632)	(12.39201, 99.896811)	(12.39168, 99.89626)
P13	7	(12.392016, 99.896703)	(12.39207, 99.89675)	(12.39178, 99.896208)	(12.39174, 99.89622)	(12.39186, 99.89629)	(12.39167, 99.89622)	(12.391892, 99.896729)	(12.39169, 99.89626)
P13	8	(12.391948, 99.897724)	(12.39199, 99.89659)	(12.391958, 99.897684)	(12.39197, 99.89768)	(12.39196, 99.89772)	(12.39197, 99.89765)	(12.39202, 99.897813)	(12.39192, 99.89766)
P13	9	(12.390928, 99.898553)	(12.39132, 99.89821)	(12.390982, 99.898581)	(12.39106, 99.89846)	(12.39099, 99.89852)	(12.39093, 99.89857)	(12.390975, 99.89852)	(12.39091, 99.89856)

Appendix ll *(Continued)*

Polygon	ID	Location (latitude, longitude)							
		1	2	3	4	5	6	7	8
P13	10			(12.390356, 99.898812)					
P13	11			(12.390961, 99.898561)					
P13	12					(12.39063, 99.90862)		(12.390622, 99.897826)	
P13	13					(12.3906, 99.89773)		(12.390618, 99.897871)	
P13	14					(12.39059, 99.89772)		(12.390622, 99.897826)	
P13	15					(12.39059, 99.89773)			
P13	16					(12.39204, 99.89769)			
P13	17						(12.39097, 99.8986)		(12.3909, 99.89857)
P13	18						(12.39088, 99.89868)		
P21	1	(12.387519, 99.909087)		(12.387889, 99.908682)		(12.387469, 99.909243)		(12.388036, 99.909252)	(12.38744, 99.90923)
P21	2	(12.387534, 99.909051)		(12.387168, 99.909263)	(12.38727, 99.90924)	(12.38726, 99.90978)	(12.38723, 99.90925)	(12.387233, 99.909249)	(12.38749, 99.90923)
P21	3	(12.388153, 99.909345)		(12.388175, 99.909339)	(12.38789, 99.90923)	(12.38757, 99.90926)		(12.388153, 99.909285)	
P21	4	(12.386922, 99.908748)		(12.387154, 99.909215)				(12.386933, 99.908835)	(12.38693, 99.90883)

Appendix ll *(Continued)*

Polygon	ID	Location (latitude, longitude)							
		1	2	3	4	5	6	7	8
P21	5	(12.38877, 99.908508)			(12.38836, 99.90857)	(12.388599, 99.908572)	(12.38769, 99.90858)	(12.388617, 99.908532)	
P21	6	(12.389275, 99.908591)		(12.389152, 99.908623)	(12.3893, 99.90874)	(12.38873, 99.90849)	(12.38926, 99.90856)	(12.38882, 99.909045)	
P21	7	(12.389503, 99.910879)		(12.389758, 99.910876)	(12.38978, 99.91074)	(12.38958, 99.91086)	(12.39049, 99.91236)	(12.389733, 99.910098)	(12.38973, 99.9101)
P21	8		(12.38921, 99.90859)			(12.388763, 99.909105)		(12.389042, 99.908996)	(12.38896, 99.90893)
P21	9		(12.38921, 99.90859)			(12.388763, 99.909105)		(12.389042, 99.908996)	(12.38896, 99.90893)
P21	10		(12.38921, 99.90859)			(12.388763, 99.909105)		(12.389042, 99.908996)	(12.38896, 99.90893)
P21	11		(12.38921, 99.90859)			(12.388763, 99.909105)		(12.389042, 99.908996)	(12.38896, 99.90893)
P21	12		(12.38921, 99.90859)			(12.388763, 99.909105)		(12.389042, 99.908996)	(12.38896, 99.90893)
P21	13		(12.38921, 99.90859)			(12.388763, 99.909105)		(12.389042, 99.908996)	(12.38896, 99.90893)
P21	14		(12.38921, 99.90859)			(12.388763, 99.909105)		(12.389042, 99.908996)	(12.38896, 99.90893)
P21	15		(12.38921, 99.90859)			(12.388763, 99.909105)		(12.389042, 99.908996)	(12.38896, 99.90893)
P21	16					(12.388763, 99.909105)		(12.389042, 99.908996)	(12.38896, 99.90893)
P21	17					(12.388763, 99.909105)		(12.388763, 99.909105)	(12.38904, 99.909)

Appendix ll *(Continued)*

Polygon	ID	Location (latitude, longitude)							
		1	2	3	4	5	6	7	8
P21	18					(12.388763, 99.909105)		(12.388763, 99.909105)	(12.38904, 99.909)
P21	19							(12.389042, 99.909)	(12.38896, 99.90893)
P21	20							(12.389042, 99.909)	(12.38876, 99.90911)
P21	21							(12.389042, 99.909)	(12.38876, 99.90911)
P21	22							(12.389042, 99.909)	(12.38876, 99.90911)
P21	23			(12.387396, 99.910689)				(12.387421, 99.910799)	
P21	24				(12.38838, 99.90862)				
P21	25				(12.38838, 99.90862)				
P21	26				(12.38838, 99.90862)				
P21	27				(12.38836, 99.90858)				
P21	28				(12.38836, 99.90858)				
P21	29				(12.38836, 99.90858)				
P21	30							(12.387421, 99.9108)	

Appendix ll *(Continued)*

Polygon	ID	Location (latitude, longitude)							
		1	2	3	4	5	6	7	8
P21	31							(12.387487, 99.91082)	
P22	1	(12.385541, 99.906099)							
P22	2	(12.386287, 99.905994)							
P22	3	(12.386425, 99.902775)		(12.386147, 99.905482)		(12.386424, 99.905534)	(12.38636, 99.90548)	(12.386408, 99.905528)	(12.38638, 99.90554)
P22	4		(12.38431, 99.90432)	(12.384286, 99.904396)	(12.38416, 99.90449)				(12.38414, 99.90447)
P22	5		(12.38418, 99.90531)						(12.38412, 99.90569)
P22	6		(12.38428, 99.90528)		(12.38409, 99.90561)				(12.38412, 99.90569)
P22	7		(12.38504, 99.90577)					(12.384529, 99.90618)	
P22	8			(12.384649, 99.904077)		(12.384641, 99.904052)			
P22	9			(12.384649, 99.904077)		(12.384641, 99.904052)		(12.384692, 99.904053)	
P22	10			(12.38523, 99.905881)	(12.38566, 99.90611)				(12.38554, 99.90597)
P22	11			(12.385702, 99.906121)	(12.38566, 99.90611)				(12.38554, 99.90597)
P22	12			(12.385747, 99.905404)	(12.38574, 99.90552)	(12.385782, 99.905522)	(12.38585, 99.9055)	(12.38576, 99.90547)	(12.38585, 99.90555)

Appendix ll *(Continued)*

Polygon	ID	Location (latitude, longitude)							
		1	2	3	4	5	6	7	8
P22	13				(12.38467, 99.90407)				
P22	14				(12.3845, 99.90622)		(12.38448, 99.90621)		(12.38447, 99.9062)
P22	15				(12.3845, 99.90619)		(12.38448, 99.90621)		(12.38447, 99.9062)
P22	16				(12.38446, 99.90617)				
P22	17				(12.38444, 99.90621)		(12.38448, 99.90621)	(12.384453, 99.906094)	(12.38447, 99.9062)
P22	18				(12.38566, 99.90611)				
P22	19					(12.385771, 99.905514)	(12.3863, 99.90507)		
P22	20					(12.383061, 99.902385)	(12.3863, 99.90507)	(12.385719, 99.905066)	(12.38586, 99.90547)
P22	21							(12.384529, 99.90618)	
P22	22								(12.38509, 99.90587)
P23	1	(12.383424, 99.903014)		(12.382649, 99.902843)				(12.382715, 99.903012)	
P23	2	(12.38374, 99.902965)		(12.383545, 99.902724)					
P23	3	(12.383587, 99.902775)	(12.38359, 99.90254)	(12.383551, 99.902736)		(12.38358, 99.90257)		(12.383599, 99.902645)	(12.38361, 99.90256)

Appendix ll *(Continued)*

Polygon	ID	Location (latitude, longitude)							
		1	2	3	4	5	6	7	8
P23	4	(12.382745, 99.902974)		(12.382649, 99.902843)				(12.382713, 99.903004)	
P23	5	(12.381485, 99.902352)		(12.381492, 99.902341)	(12.38146, 99.90235)	(12.38147, 99.9024)	(12.38148, 99.90249)		(12.3815, 99.90258)
P23	6	(12.38161, 99.903368)		(12.381515, 99.902974)					
P23	7	(12.385541, 99.903933)		(12.381515, 99.902974)	(12.38165, 99.90398)	(12.38165, 99.90398)	(12.38158, 99.90391)	(12.38166, 99.903977)	
P23	8	(12.381642, 99.903933)		(12.381515, 99.902974)		(12.381584, 99.903913)	(12.3816, 99.90394)	(12.381615, 99.903934)	
P23	9		(12.38348, 99.90371)		(12.38355, 99.90275)		(12.38345, 99.90305)	(12.383424, 99.903074)	
P23	10		(12.38348, 99.90371)						
P23	11		(12.38362, 99.90246)						
P23	12		(12.38362, 99.90246)						
P23	13		(12.38362, 99.90246)						
P23	14			(12.382927, 99.904054)					
P23	15			(12.383048, 99.902282)	(12.38367, 99.9024)	(12.383061, 99.902385)			
P23	16			(12.381511, 99.903122)	(12.38143, 99.90313)		(12.38149, 99.90311)		(12.38151, 99.90306)

Appendix ll *(Continued)*

Polygon	ID	Location (latitude, longitude)							
		1	2	3	4	5	6	7	8
P23	17			(12.381934, 99.902498)	(12.38194, 99.90249)	(12.381902, 99.902474)	(12.38154, 99.90263)	(12.381932, 99.902474)	(12.38194, 99.90246)
P23	18			(12.381934, 99.902498)	(12.38194, 99.90248)	(12.381902, 99.902474)	(12.38154, 99.90263)	(12.381932, 99.902474)	(12.38194, 99.90246)
P23	19			(12.381934, 99.902498)	(12.38194, 99.90248)	(12.381902, 99.902474)	(12.38155, 99.90261)	(12.381932, 99.902474)	(12.38194, 99.90246)
P23	20			(12.381508, 99.902357)					
P23	21			(12.381662, 99.903923)		(12.381618, 99.90393)	(12.3816, 99.90394)		
P23	22				(12.38367, 99.9024)				
P23	23				(12.38359, 99.90241)				(12.38362, 99.90259)
P23	24				(12.38363, 99.90242)				(12.38362, 99.90259)
P23	25					(12.381584, 99.903913)		(12.381654, 99.903898)	
P23	26						(12.38264, 99.90266)		
P23	27							(12.381548, 99.90396)	
P23	28								(12.38362, 99.90259)
P23	29								(12.38362, 99.90259)

Appendix ll *(Continued)*

Polygon	ID	Location (latitude, longitude)							
		1	2	3	4	5	6	7	8
P23	30								(12.38362, 99.90259)
P31	1	(12.376929, 99.889204)						(12.376872, 99.889391)	
P31	2	(12.376908, 99.889345)						(12.376903, 99.889417)	
P31	3	(12.377026, 99.889567)			(12.37716, 99.88959)			(12.377001, 99.889297)	
P31	4	(12.377712, 99.893731)				(12.377689, 99.890317)			
P31	5	(12.378001, 99.893657)							
P31	6	(12.377903, 99.893731)							
P31	7		(12.37658, 99.88994)	(12.376577, 99.889935)					(12.376573, 99.890043)
P31	8		(12.37754, 99.88995)						
P31	9		(12.37754, 99.88995)						
P31	10		(12.37934, 99.89202)		(12.37917, 99.89212)	(12.378621, 99.891309)	(12.37895, 99.89256)		(12.379388, 99.892114)
P31	11		(12.37772, 99.8937)						
P31	12		(12.37822, 99.89393)		(12.37776, 99.89399)			(12.377678, 99.894113)	

Appendix ll *(Continued)*

Polygon	ID	Location (latitude, longitude)							
		1	2	3	4	5	6	7	8
P31	13		(12.37759, 99.894)		(12.37777, 99.89388)	(12.377674, 99.893803)			(12.38362, 99.90259)
P31	14		(12.37757, 99.894)		(12.37777, 99.89388)				
P31	15		(12.37754, 99.89403)						
P31	16		(12.37751, 99.89403)		(12.37777, 99.89388)				
P31	17		(12.37755, 99.89404)						
P31	18		(12.37755, 99.89404)						
P31	19				(12.37817, 99.89364)				
P31	20				(12.37651, 99.89041)				(12.376573, 99.890043)
P31	21				(12.37651, 99.89041)				
P31	22				(12.37651, 99.89041)				
P31	23				(12.37656, 99.89067)				(12.379388, 99.892114)
P31	24				(12.37685, 99.88947)				
P31	25					(12.376861, 99.891298)			

Appendix ll *(Continued)*

Polygon	ID	Location (latitude, longitude)							
		1	2	3	4	5	6	7	8
P31	26					(12.378573, 99.891277)		(12.378777, 99.8912)	
P32	1		(12.37975, 99.89562)						
P32	2		(12.38082, 99.89595)		(12.37956, 99.89595)				
P32	3					(12.379139, 99.899793)	(12.37954, 99.89591)		
P32	4					(12.379114, 99.899747)			
P32	5					(12.379219, 99.899788)			
P32	6						(12.37772, 99.89973)		
P32	7						(12.37778, 99.89973)		
P32	8						(12.37775, 99.89972)		
P33	1	(12.38544, 99.900526)							
P33	2	(12.384879, 99.899603)	(12.3848, 99.89931)	(12.384817, 99.899373)	(12.38474, 99.89923)	(12.38477, 99.8998)	(12.38467, 99.89929)		(12.384043, 99.89961)
P33	3		(12.38491, 99.89698)						
P33	4		(12.38491, 99.89698)	(12.384727, 99.898198)					

Appendix ll *(Continued)*

Polygon	ID	Location (latitude, longitude)							
		1	2	3	4	5	6	7	8
P33	5		(12.38491, 99.89698)						
P33	6		(12.38491, 99.89698)						
P33	7		(12.38491, 99.89698)						
P33	8		(12.38491, 99.89698)						
P33	9		(12.38491, 99.89698)						
P33	10			(12.38393, 99.899712)					
P33	11			(12.383903, 99.899908)					
P33	12			(12.385723, 99.898484)					
P33	13			(12.385723, 99.898484)					
P33	14			(12.385665, 99.90029)					
P33	15						(12.38611, 99.89953)		
P33	16						(12.38466, 99.89997)		
P33	17						(12.38471, 99.89981)		

Appendix ll *(Continued)*

Polygon	ID	Location (latitude, longitude)							
		1	2	3	4	5	6	7	8
P41	1	(12.377277, 99.903428)							
P41	2	(12.377172, 99.903572)	(12.37713, 99.9037)	(12.377415, 99.90295)					
P41	3	(12.377915, 99.90286)	(12.37734, 99.90324)		(12.37745, 99.90313)				
P41	4	(12.37795, 99.902283)				(12.377386, 99.903221)		(12.377817, 99.902312)	(12.377805, 99.902295)
P41	5	(12.377718, 99.90278)		(12.377555, 99.902927)					
P41	6	(12.377705, 99.902583)							
P41	7		(12.37724, 99.90366)		(12.377, 99.90389)				
P41	8		(12.37882, 99.91033)		(12.37777, 99.90222)	(12.377826, 99.902171)		(12.377817, 99.902312)	(12.377796, 99.902369)
P41	9			(12.377418, 99.903073)					
P41	10			(12.37742, 99.903127)					
P41	11				(12.37704, 99.90391)				
P42	1		(12.38287, 99.90613)						
P42	2		(12.38295, 99.90621)						

Appendix ll *(Continued)*

Polygon	ID	Location (latitude, longitude)							
		1	2	3	4	5	6	7	8
P42	3		(12.38244, 99.90839)						
P42	4			(12.382312, 99.90672)				(12.382411, 99.907167)	
P42	5			(12.381867, 99.907565)					
P42	6			(12.382267, 99.907965)					
P42	7			(12.383868, 99.908855)		(12.383515, 99.908963)			
P42	8			(12.383877, 99.908845)				(12.383969, 99.908907)	
P42	9					(12.383657, 99.907112)		(12.383657, 99.907112)	
P43	1	(12.377022, 99.907563)	(12.3771, 99.90722)		(12.37696, 99.9074)	(12.37697, 99.90739)	(12.37915, 99.90666)		(12.37697, 99.907715)
P43	2	(12.378852, 99.908008)		(12.3789, 99.907732)	(12.37871, 99.90782)				(12.376969, 99.907389)
P43	3	(12.378768, 99.90758)	(12.37914, 99.90666)	(12.377802, 99.907173)	(12.37868, 99.90782)				
P43	4	(12.378823, 99.90754)							
P43	5	(12.378922, 99.907243)	(12.379, 99.90709)	(12.37887, 99.907217)		(12.37915, 99.90666)		(12.37911, 99.907051)	(12.37911, 99.907051)
P43	6	(12.378897, 99.907219)		(12.379117, 99.907233)		(12.378973, 99.907201)		(12.378895, 99.907372)	

Appendix ll *(Continued)*

Polygon	ID	Location (latitude, longitude)							
		1	2	3	4	5	6	7	8
P43	7	(12.379203, 99.907106)				(12.379261, 99.907217)			
P43	8		(12.37704, 99.90745)	(12.377027, 99.907502)	(12.37692, 99.90766)	(12.37692, 99.90789)		(12.37702, 99.907503)	(12.37702, 99.907493)
P43	9			(12.38025, 99.906935)					
P43	10				(12.378, 99.90849)		(12.37796, 99.90817)		(12.37778, 99.90871)
P43	11				(12.37779, 99.9088)		(12.37796, 99.90817)		
P43	12				(12.378, 99.90849)		(12.37796, 99.90817)		
P43	13				(12.37945, 99.9066)				
P43	14				(12.37697, 99.9077)	(12.376841, 99.908028)			
P43	15				(12.37691, 99.90786)	(12.37688, 99.907988)	(12.37915, 99.90699)		
P43	16					(12.376865, 99.908024)			
P43	17					(12.378268, 99.906955)			
P43	18					(12.379261, 99.907217)			
P43	19					(12.379261, 99.907217)		(12.37948, 99.907154)	

Appendix ll *(Continued)*

Polygon	ID	Location (latitude, longitude)							
		1	2	3	4	5	6	7	8
P43	20					(12.379403, 99.907159)		(12.379514, 99.907216)	
